# Identification of cellulolytic lactic acid bacteria from the intestines of laying hens given AKBISprob based on 16S ribosomal ribonucleic acid gene analysis

**DOI:** 10.14202/vetworld.2022.1650-1656

**Published:** 2022-07-14

**Authors:** Nurliana Nurliana, Baharuddin Halim Siregar, Wahyu Eka Sari, Teuku Zahrial Helmi, Sugito Sugito

**Affiliations:** 1 Laboratory of Veterinary Public Health, Faculty of Veterinary Medicine, Universitas Syiah Kuala, Banda Aceh, Indonesia; 2 Study Program of Veterinary Education, Faculty of Veterinary Medicine, Universitas Syiah Kuala, Banda Aceh, Indonesia; 3 Laboratory of Research, Faculty of Veterinary Medicine, Universitas Syiah Kuala, Banda Aceh, Indonesia; 4 Laboratory of Biochemical, Faculty of Veterinary Medicine, Universitas Syiah Kuala, Banda Aceh, Indonesia; 5 Laboratory of Clinic, Faculty of Veterinary Medicine, Universitas Syiah Kuala, Banda Aceh, Indonesia

**Keywords:** 16S ribosomal ribonucleic acid, AKBISprob, *Enterococcus*, phylogenetic tree, polymerase chain reaction

## Abstract

**Background and Aim:**

Supplementation of AKBISprob (developed in a previous study) in feed can improve production efficiency and poultry health, especially laying hens. In addition, it can also increase cellulolytic lactic acid bacteria (LAB) in chicken intestines, but these bacteria are still unknown; thus, they need to be identified. This study aimed to identify cellulolytic LAB in the intestines of laying hens administered AKBISprob based on 16S ribosomal ribonucleic acid (16S rRNA) gene analysis.

**Materials and Methods:**

The samples used in this study were 13 LAB isolates from the intestines of laying hens that were given AKBISprob 4%. Cellulolytic LAB DNA was isolated and 16S rRNA gene was amplified by polymerase chain reaction, followed by sequencing, bioinformatics analysis, and phylogenetic tree construction.

**Results:**

From 10 cellulolytic LAB isolates with a clear zone of >6 mm, four were selected and their DNA was amplified with BaCF and UniB primers ~1500 bp DNA fragments. Of these, the P31H62 isolate was genetically close to *Enterococcus hirae* strain 1-1X-16 with 92.90% maximum identity, the P33S52 isolate had homology with *Enterococcus mundtii* strain ZU 26 with 96.76% maximum identity, and the P33S62 isolate was closely related to *E. hirae* strain SJ3 with 72.96% maximum identity. The phylogenetic tree revealed that the cellulolytic LAB isolates P31H62 and P33S52 were in one cluster closely related to the genus *Enterococcus*.

**Conclusion:**

This study suggests that the isolates P31H62, P33S62, and P33S52 from the intestines of laying hens administered 4% AKBISprob are cellulolytic LAB belonging to the genus *Enterococcus*.

## Introduction

Lactic acid bacteria (LAB) are a group of Gram-positive bacteria that are round or rod-shaped and catalase-negative, do not form spores [1], and can ferment sugars or carbohydrates to produce large amounts of lactic acid [2]. Some active metabolites produced by LAB are lactic acid, ethanol, hydrogen peroxide, and bacteriocin [3, 4]. LAB are generally recognized as safe [2]; thus, they can be used as probiotic agents. According to Bairagi *et al*. [5], probiotic bacteria have amylolytic, proteolytic, lipolytic, and cellulolytic activities. LAB produce not only lactic acid and bacteriocin but also several extracellular enzymes, such as amylase, pectinase, β-glucosidase, and cellulase [6]. Chickens have difficulty absorbing feed containing crude fiber, such as cellulose; thus, the presence of cellulolytic bacteria in their intestines is important for digestion. The use of probiotics or prebiotics together with live *Salmonella* vaccination is an excellent way to reduce the adverse effects of harmful bacteria and improve the growth performance of broilers [7].

Cellulolytic bacteria can degrade cellulose-containing substrates. Cellulolytic bacteria can convert cellulose into sugar, which can then be used as a carbon source and an energy source for metabolism and growth [8, 9]. Cellulose-producing microbes from bacteria have an advantage over fungi in that they grow faster and thus require less time to produce cellulase [10]. Research on cellulolytic bacteria has been widely conducted, and the findings have been applied in various fields, including animal husbandry and industry. In animal husbandry, the use of cellulolytic *Acetobacter liquefaciens* species effectively increases daily weight gain and decreases animal feed conversion rates [11]. *Lactobacillus* spp., such as *Lactobacillus plantarum* [12] and *Levilactobacillus brevis* [13], are known to have cellulolytic activity. One way to increase LAB population in chicken intestines is by supplementing their feed with symbiotics [14]. Nurliana *et al*. [15] developed an alternative modification of fermented poultry feed, which is a mixture of mule pulp and palm kernel cake with the addition of *Aspergillus niger*-type microbes called AKBISprob. The addition of AKBISprob at a dose of 4% in feed can improve production efficiency and health in poultry, specifically, laying hens and broilers. AKBISprob supplementation can also increase cellulolytic LAB in chicken intestines [15], but the species of cellulolytic LAB are still unknown; thus, they need to be identified.

Bacterial identification can be made based on phenotypic and genetic characteristics [16, 17]. By sorting bases from bacterial DNA, genetic methods for bacterial identification and characterization provide fast and accurate results [18]. The genetic and molecular identification of bacteria is generally conducted using 16S ribosomal ribonucleic acid (16S rRNA). The 16S rRNA gene is commonly used for identifying prokaryotic species through polymerase chain reaction (PCR) [19–21]. PCR can duplicate small amounts of target DNA, even if a single strand of DNA is used. PCR is a very sensitive, specific, and fast method [22].

Based on this background, this study aimed to identify cellulolytic LAB species in chicken intestines treated with AKBISprob based on 16S rRNA gene analysis using a PCR.

## Materials and Methods

### Ethical approval

There is no need for ethical approval because this study did not use chicken as the sample but used lactic acid bacteria isolates that had been collected previously.

### Study period and location

This research was conducted from January to June 2019 at the Laboratory of Veterinary Public Health and Laboratory of Research, Faculty of Veterinary Medicine, Universitas Syiah Kuala. The sequencing process was carried out at 1^st^ BASE Laboratories, Malaysia.

### Research design

This research was conducted in a laboratory in a series of steps. The first step was to select cellulolytic LAB on De Man, Rogosa and Sharpe (MRS) agar (Oxoid Ltd., United Kingdom) plus carboxymethylcellulose (CMC) (Oxoid Ltd). The next step was DNA isolation, followed by PCR amplification of the 16S rRNA gene, sequencing, and homology analysis of DNA sequences. The final step was to construct a phylogenetic tree.

### Screening of cellulolytic LAB

LAB isolates stored in MRS broth (Oxoid Ltd) were centrifuged for 3 min at 14,000–16,000× *g*. Cellulase detection was based on the method proposed by Nurliana *et al*. [23]. The agar diffusion method employed 0.7% MRS agar (Oxoid Ltd) and 0.5% CMC in distilled water, which was autoclaved at 121°C for 15 min. The media was poured into a Petri dish and allowed to solidify, and then the blank disk paper was dipped in 25 uL of the LAB isolate supernatant and left for 15 minutes. Next step, the disk paper dipped with the LAB supernatant was placed on top of the solid media and the medium was incubated for 24 h at 37°C. On observation of cellulolytic activity, the medium was soaked for 15 min with 0.1% Congo red (Merck, Germany) solution in 70% alcohol, after which 1 M NaCl was poured for 15 min. Isolates with clear zones indicated the presence of cellulolytic processes by LAB. LAB isolates with the four largest clear zones were transferred to MRS broth for further examination.

### DNA isolation of cellulolytic LAB

Genomic DNA isolation of cellulolytic LAB was performed using the Presto™ Mini gDNA Bacteria Kit (Geneaid Ltd., Taiwan). Then, 1.5 mL (1 × 10^9^ cells/mL) of MRS broth was transferred into a 1.5 mL tube, and the microtubes were centrifuged for 1 min at 14,000–16,000× *g*. The supernatant was discarded, leaving behind the pellets in the tube. Gram-positive buffer (200 μL) with lysozyme (0.8 mg/mL) was added to the pellets and homogenized with a vortex. Proteinase K (20 μL) was added, and the solution was incubated for 10 min at 60°C and inversed every 3 min. The cell lysis process included the addition of Gram-positive buffer (200 μL) to the sample, homogenization for 10 s, and incubation at 70°C for 10 min. The solution was inversed every 3 min. The cell-binding process involved the addition of absolute ethanol (200 μL) followed by homogenization. The GD column was moved into a 2 mL tube and centrifuged at 14,000–16,000× *g* for 2 min, and the supernatant was discarded. Then, the GD column was transferred to a new 2 mL collection tube. The washing process included the addition of W1 buffer (400 μL) and centrifugation for 3 min, after which the supernatant was discarded. Wash buffer (600 μL) and ethanol mixture were added, after which the solution was centrifuged at 14,000–16,000× *g* for 3 min and the supernatant was discarded. The GD column was transferred to a new 2 mL tube, and elution buffer (30 μL) was added (heated at 70°C). The solution was incubated at room temperature (27–29°C) for 3–5 min and centrifuged at 14,000–16,000× *g* for 1 min. The tube with DNA was stored at −20°C. The final step was to isolate DNA through electrophoresis using a 1% agarose gel for 60 min at 60 V in comparison with the samples inserted into the electrophoresis well (1 μL loading dye/5 μL sample). After the migration process was complete, agarose gel was visualized using GelDoc (Bio-Rad, USA). The success of the DNA isolation was characterized by the presence of good-quality DNA bands on the electrophoresis gel (Bio-Rad)..

### PCR amplification of the 16S rRNA gene

The PCR mixture contained 3.5 μL nuclease-free water, 12.5 μL KAPA2G Fast ReadyMix, 2 μL of 10 μM forward primer (BaCF), 2 μL of 10 μM reverse primer (UniB), and 5 μL DNA templates, for a total volume of 25 μL inserted into the PCR tube. PCR amplification included pre-denaturation at 94°C for 3 min, denaturation at 94°C for 1 min, annealing at 57°C for 1 min, extension at 72°C for 1 min, and post-extension at 72°C for 5 min. PCR products were electrophoresed on a 1% agarose gel for 60 min at 60 V, and a single DNA band was observed on GelDoc to ensure that DNA fragments were amplified at a size of ~1500 bp.

### Sequencing, bioinformatics analysis, and phylogenetic tree construction

The sequencing process was performed using the commercial services of 1^st^ BASE (Malaysia). The nucleotide sequence results were compared with GenBank using BLASTN from the National Center for Biotechnology Information (NCBI) (http://www.ncbi.nih.gov.). Alignment of DNA sequences to classify genes of the same size and phylogenetic tree construction of 16S rRNA genes were conducted using MEGA 5.05 [24] based on the neighbor-joining tree method, with a bootstrap value of 1000×.

## Results

### Screening of cellulolytic LAB

The formation of a clear zone around the paper disk on MRS agar plus CMC indicates that LAB are cellulolytic. A semi-quantitative analysis of cellulolytic bacterial activity was performed by measuring the clear zones around the colony. The clear zones of 13 LAB isolates from the intestines of laying hens were measured (Table-1).

**Table 1:** Clear zone diameters of 13 lactic acid bacteria isolates at MRSA 0.7% and CMC 0.5%.

Isolates	Diameters of clear zone (mm)
P11H62	4.18
P12H52	1.86
P12S73	4.75
P21H71	1.86
P23H31	3.35
P31H54	12.15
P31H62	7.86
P31S51	5.97
P31S71	-
P31S72	-
P31S51	-
P33S52	6.27
P33S62	6.28

The measurement results of the clear zones of the 13 tested LAB isolates indicated that 10 LAB isolates had a cellulolytic activity with different clear zone diameters. Based on the clear zone diameters of 10 cellulolytic LAB isolates, four selected isolates with the largest clear zone diameter were obtained: P31H54 with a diameter of 12.15 mm, P31H62 with 7.86 mm, P33S52 with 6.27 mm, and P33S62 with 6.28 mm. Identification of 16S rRNA genes from cellulolytic LAB isolates was performed. The P31H54 isolate had the highest cellulolytic activity compared with other isolates, indicating that P31H54 had the greatest ability to digest fiber.

### Morphological characteristics of cellulolytic LAB

**Figure-1:**
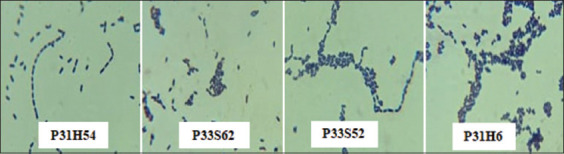
Gram staining results of four lactic acid bacteria 1000× cellulolytic isolates.

Cellulolytic LAB were identified by macroscopic and microscopic morphological observations. Size, shape, elevation, edges, and color were among the examined morphological features of the cellulolytic LAB colonies (Table-2). Macroscopically, colonies of the four cellulolytic LAB isolates were round and had a convex elevation, a smooth or flat texture, colored colonies (creamy or white color), and an average size of 2 mm. Microscopically, the P31H54 isolates were rod or bacillus shaped, while the P31H62, P33S52, and P33S62 isolates were short rod or coccobacillus shaped. The Gram staining results are presented in Figure-1.

**Table 2:** Macroscopic description of morphological colonies of cellulolytic lactic acid bacteria isolates.

Isolates	Size (mm)	Elevation	Edge	Color
P31H54	2.05	Convex	Slippery	Cream
P31H62	2.23	Convex	Slippery	Cream
P33S52	2.02	Convex	Slippery	Cream
P33S62	2.07	Convex	Slippery	Cream

### DNA genome of cellulolytic LAB

**Figure-2:**
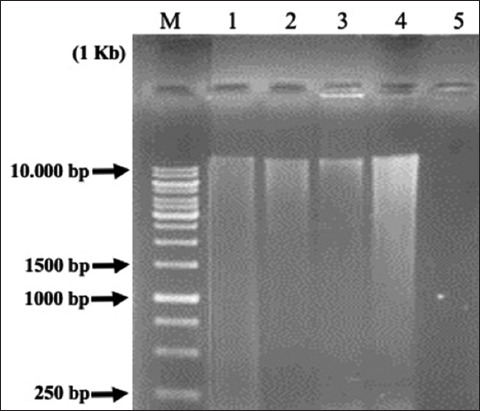
Visualization results of cellulolytic lactic acid bacteria DNA isolation on the 1% agarose gel. 1 Kb = Marker; 1 = P31H62; 2 = P33S52; 3 = P33S62; and 4 = P31H54.

The cellulolytic LAB genome isolation results obtained from the four samples are presented in Figure-2. The DNA band formation in agarose gels showed the migration pathway of DNA with varying band thicknesses. As a result, the DNA samples had varying qualities and quantities.

### 16S rRNA gene of cellulolytic LAB

**Figure-3:**
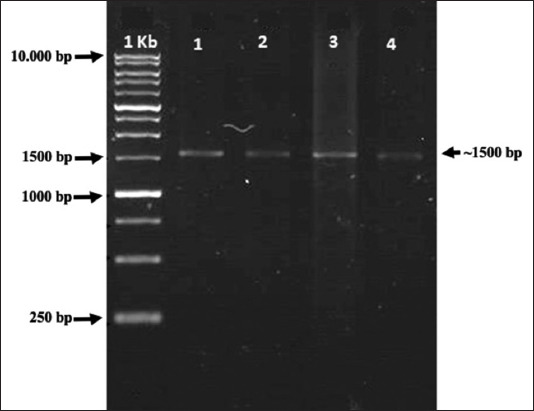
Polymerase chain reaction amplification of 16S ribosomal ribonucleic acid gene from lactic acid bacteria cellulolytic isolates ~ 1500 bp. 1 Kb (Marker); 1 = P31H62; 2 = P33S52; 3 = P33S62; and 4 = P31H54.

Based on the amplification results, the DNA isolate sample is presented in Figure-3. The obtained results are parallel DNA bands on the marker at ~1500 bp. Using BaCF and UniB primers, the four cellulolytic LAB isolates had successfully amplified 16S rRNA genes, with expected DNA fragment sizes of ~1500 bp.

### Bioinformatics analysis and genetic relationships of the 16S rRNA gene of cellulolytic LAB

Based on the BLAST analysis of 16S rRNA partial sequences from four parallel isolates, the P31H62 isolate had similarities with *Enterococcus hirae* strain 1-1X-16 (92.90%), the P33S52 isolate had similarities with *Enterococcus mundtii* strain ZU 26 (96.76%), the P31H54 isolate had similarities with *Bacillus mycoides* strain HB55 (91.03%), and the P33S62 isolate had similarities with *E. hirae* strain SJ3 (72.96%; Table-3). The P31H62, P33S52, and P31H54 isolates had identity values of <97% with an E = 0.0, whereas the P33S62 isolate had an E > 0. The resulting E = 0 indicated a sequence of homologous samples with GenBank sequences. The most similar GenBank sequences were characterized by the same max score and total score; the query coverage was close to 100%, the E-value was close to 0.0, and the max identity was close to 100%.

**Table 3:** Similarity of cellulolytic lactic acid bacteria 16S ribosomal ribonucleic acid gene sequences with GenBank.

Isolates	References strain (GenBank)	% Similarity	Accession number
P31H62	*Enterococcus hirae strain* 1-1X-16	92.90	CP015516.1
P33S52	*Enterococcus mundtii strain* ZU 26	96.76	AB548881.1
P33S62	*Enterococcus hirae strain* SJ3	72.96	MG966462.1
P31H54	*Bacillus mycoides strain* HB55	91.03	JX856133.1

**Figure-4:**
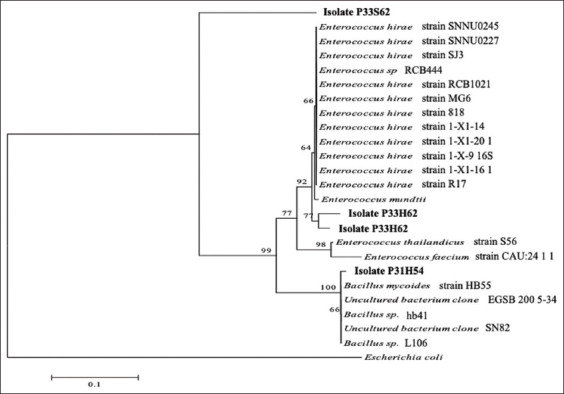
Phylogenetic tree of 16S ribosomal ribonucleic acid gene of cellulolytic lactic acid bacteria with bootstrap 1000×. Kindly mention the text in the image to the proper placement.

Based on the phylogenetic tree construction, the P31H62, P33S52, and P33S62 isolates were clustered together with the genus *Enterococcus*, whereas the P31H54 isolate was in Cluster II, which was closely related to the genus *Bacillus* (Figure-4), a group of Gram-positive bacteria. But when compared to outgroup cluster Gram-negative bacteria (*Escherichia coli*), the four isolates (P31H62, P33S52, P33S62, and P31H54) were separated from them.

Based on the phylogenetic tree construction of 16S rRNA genes from the four cellulolytic LAB isolates aligned with the isolates found in GenBank, the main branch with a bootstrap value of 100% was formed, namely, the *Bacillus* genus branch cluster with the P31H54 isolate. This demonstrated that the two branches were consistent. The P31H62 and P33S52 isolates had bootstrap values of >70% and 93%, respectively, with the genus *Enterococcus*. Although the P33S62 isolate was located far from the *Enterococcus* genus cluster, this was in accordance with the BLAST analysis result of the isolate, which was equal to 72.98%.

## Discussion

Cellulolytic LAB screening is characterized by a clear zone formation around the paper disk on MRS agar plus CMC. The addition of 0.5% CMC to MRS agar can be used as a selective medium for cellulolytic LAB [23]. Cellulolytic activity is indicated by the presence of a clear zone on CMC agar medium after being given Congo red dye. This is because no interaction between Congo red and the polysaccharides in the medium occurs. According to Gupta *et al*. [25], clear zones form on the medium soaked with 0.1% Congo red because cellulase hydrolyzes cellulose in the medium into simple compounds, namely, cellobiose, which is then simplified into two glucose molecules. Warly *et al*. [26] stated that depending on the genes present and the carbon source used, each cellulolytic bacterium can produce different cellulase complexes. The ability of each bacterial species to hydrolyze the CMC substrate to produce different cellulases accounts for the difference in the clear zone of each isolate [27]. CMC is the best substrate for inducing the synthesis of extracellular cellulolytic enzymes. Cellulolytic bacteria, which produce cellulase, can hydrolyze cellulose when it is present in their environment [26]. Cellulolytic bacteria can hydrolyze complex cellulose into simple oligosaccharides and, eventually, glucose.

Cellulolytic LAB exhibited round bacterial colonies with convex elevation, smooth or flat texture, and colored colonies (cream or white milk). This was consistent with the finding of Wiryawan *et al*. [28]; the morphology of LAB in the four samples from the digestive tract of laying hens formed milky white, yellow, and cream-colored colonies with a perfectly round shape. Gram staining is an important step in the initial identification of bacteria, which examines their morphological characteristics. Bacteria observed by Gram staining under a microscope were Gram-positive and retained the color of the crystals and displayed a purple color. According to Melia and dan Yuherman [29], LAB are Gram-positive, rod-shaped bacteria. The purple color in Gram-positive bacteria is caused by the main component of Gram-positive cell walls, peptidoglycan, which binds to the violet crystals.

In this study, four LAB isolates were successfully isolated using the Presto™ Mini gDNA Bacteria Kit, with genomic DNA of >10,000 bp length successfully isolated from all four samples. Cell lysis, also known as bacterial cell damage, is necessary for the destruction of protein structures and allows the release of nucleic acids. Because cellulolytic LAB isolates are Gram-positive bacteria with a cell wall structure that includes a thick and strong peptidoglycan layer, the bacterial cell wall can be destroyed by adding lysozyme. Lysozyme digests bacterial cell walls by hydrolyzing a thick peptidoglycan layer [30].

After DNA isolation, DNA amplification was performed using PCR. PCR amplified DNA fragments by targeting the 16S rRNA gene sequence using BaCF and UniB primers, designed to complement the continuous area in the bacterial domain and the universal sustainable region in the 16S rRNA gene of *Escherichia coli* [31]. The amplification process is the most important step. The primary form is a single, short DNA strand (oligonucleotide) that initiates the reaction of the DNA polymerization process *in vitro* and recognizes and marks the DNA fragments (templates) to be amplified [32]. This indicated that gene fragments from LAB isolates were amplified using universal primers for the amplification of 16S rRNA genes with a target of a 1500 bp amplicon. Visualization of DNA isolates revealed a single band on the GelDoc, indicating that the obtained DNA was of good quality and the 16S rRNA gene had a size of 1500–1550 bp [33].

The similarity of the DNA sequences of cellulolytic LAB isolates to the DNA sequences deposited in GenBank can be determined by aligning DNA sequence samples with nucleotide data on the NCBI (http://www.nlm.nih.gov). Alignment was performed with BLASTN [34]. Furthermore, up to five nucleotide sequences with the highest similarity to the sample were downloaded from the same website and stored in the form of FASTA (text) to facilitate editing. The 16S rRNA nucleotide sequence results of the four cellulolytic LAB isolates from “First Base” were aligned using BLASTN and found to be similar to the sequences in GenBank. The closest sequence to the sample had the highest similarity [35]. If the homology approached 100% or >97%, it could be confirmed as the same species; conversely, if the homology was <97%, the isolate was possibly a new species, or the gene could not be confirmed.

Phylogenetic tree construction is based on alignment (of DNA sequences) using the neighbor-joining tree method with a 1000× bootstrap on MEGA 5.05. According to Telles *et al*. [36], the neighbor-joining tree method is employed for phylogenetic tree reconstruction by calculating the changes in DNA bases in each organism (evolutionary distance) as visualized by the length of the branch formed. Bootstrap values at branching indicate the accuracy of branching values in phylogenetic trees, with a calculation of 1000 randomizations [37]. A bootstrap value of 100% at branching also shows that the branching is consistent [33]. Bootstrap values of ≥ 95% indicate that the branching topology is accurate and consistent or that it will not change even if performed using other phylogenetic tree preparation methods [38]. According to Coenye and Vandamme [39], a bootstrap value of >70% indicates that the branching is significant and permanent. Low bootstrap values indicate that branching will very likely change [39]. Based on phylogenetic analysis, the three isolates classified as genus *Enterococcus*, namely, P31H62, P33S62, and P33H62, were identified as LAB. The P31H54 isolate belonged to the genus *Bacillus*. LAB belong to the phylum Firmicutes, class Bacilli, order Lactobacillales, and family Lactobacillaceae and consists of 10 genera, namely, *Lactobacillus*, *Aerococcus*, *Leuconostoc*, *Pediococcus*, *Streptococcus*, *Weisella*, *Carnobacterium*, *Tetrococcus*, *Tetragenococcus*, and *Bifidobacterium* [40].

## Conclusion

This study demonstrated that the cellulolytic LAB isolates P31H62, P33S62, and P33S52 from the intestines of laying hens administered 4% AKBISprob belonged to the genus *Enterococcus*. The isolates had homology with *E. hirae* strain 1-1X-16, *E. mundtii* strain ZU 26, and *E. hirae* strain SJ3. Further study is necessary to create a bacterial consortium from the three isolates that have been identified by the LAB species as probiotic candidates in animal feed.

## Authors’ Contributions

NN, WES, and TZH: Conceptualized and designed the study. BHS, WES, and NN: Carried out the experiment. WES: Analysis of the data. NN, WES, BHS, TZH, and SS: Drafted and revised the manuscript. All authors have read and approved the final manuscript.
